# Supplementary material for: “How I wish we could manage such things”: A qualitative assessment of barriers to postpartum hemorrhage management and referral in Kenya
Source: PLOS Glob Public Health. 2024 Nov 1;4(11):e0003842. doi: 10.1371/journal.pgph.0003842 (PMC11530065; doi:10.1371/journal.pgph.0003842)
Supplement: S1 Text — (DOCX) [file pgph.0003842.s002.docx]

*S1: PHC Provider Interview Guide*

**General/Background Information (Provider):**

*First, I would like to get some general/background information on you and your facility*:

1. Age:
2. Gender:
3. Cadre:
4. Years of Professional Practice:
5. How long have you been working in this facility?
6. Length of time in your current position:
7. How many deliveries do you personally attend to per month?

**General/Background Information (Facility):**

1. How many patients are typically seen in this facility each month?
2. How many deliveries occur in this facility each month?
3. How many clinic staff are present in the facility during a typical shift? How is work distributed across clinical staff in the facility?
4. Can you please describe how the staff members in the hospital are assigned to the maternity unit?

-*Probing Questions:* Are there day and night shifts? If so, how are providers assigned to each shift? How many providers are present in each shift (day and night)? What are the differences between weekdays and weekends?

1. While you are on duty, is there any staff member that is “on call” for additional support? Under what conditions should this on-call provider be contacted? In your experience, are on-call providers available when you need to speak with them?
2. Does the facility have an in-house lab and scientist? If yes, what kind of tests are they able to do for pregnant women in the in-facility lab?

**Postpartum hemorrhage management:**

*Now I would like to ask some questions on PPH management.*

1. Can you please describe to me what kind of care women receive from delivery until they are discharged? How are they monitored?
2. What risk factors do you check for? How do you check for these?
3. What are the most common causes of PPH in this facility? Which causes do you see most frequently in your work?
4. Where are PPH cases usually found? For example, do most PPH cases happen in labor and delivery or later during post-partum care?
5. How do you typically make a diagnosis of PPH in this facility? Do you measure the blood loss? If yes, how do you measure it (e.g. measuring jar)?
6. How many cases of PPH do you think this facility has each month? Are these cases typically reviewed by staff for learning purposes?
7. What medications and supplies are available in the room when PPH is identified?
8. What medications and supplies do you usually need for managing PPH cases in this facility? How does the facility acquire those supplies? How frequently do you experience stock-outs? Where are the PPH-related supplies stored in this facility? When there is a case of PPH, how do you access the supplies from the stores?
9. Are the medications and supplies needed to treat PPH available in the delivery room? In the facility? Are they easily accessible?

**UBT Knowledge and Training**

***Now I would like to ask you some questions about the Uterine Balloon Tamponade (UBT).***

1. Have you ever heard of UBT? Where did you first hear about it?
2. What do you think about UBT?
3. What are perceptions of other providers regarding the use of UBT? What do providers say to one another about UBT?
4. Have you participated in any UBT training (other than in school, if applicable)? When?
5. Is UBT used to stop PPH in this facility? How do you decide when to use UBT/When is it used?
6. Are there any specific challenges that make it difficult to use UBT at this facility?

**PPH Referral Case:**

*Now I would like to ask you about a specific PPH case that was referred from this facility. As a reminder, this information will only be used to better understand how PPH cases are managed and referred. There are no right or wrong answers and your responses will have no impact on your job at this facility or elsewhere. Your name will not be recorded with any of your answers.*

*The case we would like to hear more about is a patient who was referred from this facility to* ***[referral facility name]*** *on* ***[date]*** *at* ***[time].***

1. Do you remember any details of this specific case? Would you please describe what happened?
2. Was the patient referred from another facility? If yes, which one? How far away is this facility (in km or in minutes of travel)?
3. What was the patient’s pregnancy status on arrival?
4. How far along in labor was she when she arrived? How quickly did she progress? Did the labor progress normally? If no, what happened?
5. What was the patient’s health condition on arrival? *Probe: Was she stable, critically ill?*
6. How was the patient monitored throughout labor and delivery?
7. What risk factors for PPH were identified, if any, for the patient?
8. Was the patient accompanied by anyone such as a husband, sister, mother-in-law, or friend?
9. When did you first notice that the patient was experiencing excessive blood loss? How did you assess if it was normal bleeding or PPH? Approximately how much blood had the patient lost when it was identified as PPH?
10. What was the cause of the PPH?
11. What treatment did you use first in order to stop the bleeding? Can you describe whether that worked or not? Did you use any other treatments/interventions?
12. After identifying the PPH, did you contact anyone to determine which treatment was necessary?
13. At what point did you decide that the patient should be referred to another facility?
14. Did you consult with anyone else at this facility about whether or not the patient should be referred?
15. Did the patient (or the support person, if present) have any concerns about being referred to another facility? If yes, can you tell me more about that?
16. How did you decide which facility to refer her to?
17. What was the name of the referral facility where you decided to send the patient?
18. How far is that facility in kilometers (*probe if they cannot respond in km: How many minutes does it take to arrive at that facility*?)
19. Please tell me how you prepare the patient for referral, if any preparation was involved. Please describe any coordination with the receiving facility, if any.
20. Did you contact the referral facility before transferring the patient? What information was shared at that time?
21. How was the patient transferred to the referral facility? Did the patient have to pay any money for this transfer?
22. Did any staff from this facility or the patient’s support person travel with the patient to the referral facility?
23. When the patient arrived at the referral facility, did you or any other staff from this facility speak with anyone at the referral facility either in-person or by phone? What information was shared at that time?
24. In total, for how many hours was the patient receiving care from this facility starting from the time that she arrived until she arrived at the referral facility?
25. After the woman was treated at the referral facility, did any staff from this facility follow-up by phone or in-person to hear how the case was managed? If yes, what information was shared at that time?
26. Do you know what treatment/intervention was used at the referral facility?
27. Do you know if the referral facility used UBT to treat the patient’s PPH?
28. Do you think this was an appropriate case for using UBT?

*If yes:* Would you tell us more about why you chose not to use UBT in this case?

*If no:* Can you tell us why you did not think UBT was appropriate in this case?

1. When this patient was experiencing PPH in this facility, was there any one on duty with experience using UBT?
2. When this patient was experiencing PPH in this facility, was there anyone whom you could call who had experience using UBT?
3. Was the patient transferred to any other facilities after the referral we just discussed?

Before we move on to other questions, is there anyone else from this facility that assisted in this specific PPH case either by providing care or advice in person or over the phone? If so, can you please provide their names so that we might include them in this study?

| Please list the name and qualifications of all providers involved in this case. Include any provider who was directly involved in patient care or who provided input (in person or over the phone) into decision-making about patient care. | |
| --- | --- |
| Provider 1: QUALIFICATION | Provider 1: NAME |
| Provider 2: QUALIFICATION | Provider 2: NAME |
| Provider 3: QUALIFICATION | Provider 3: NAME |
| Provider 4: QUALIFICATION | Provider 4: NAME |
| Provider 5: QUALIFICATION | Provider 5: NAME |
| Provider 6: QUALIFICATION | Provider 6: NAME |

**Referral Process**

***Now I would like to ask you some questions about the referral process in general.***

1. About how many maternity cases do you refer per month? What are the most common reasons for referral?
2. Who typically makes decisions about referrals? Is that different if it is daytime versus nighttime?
3. How are referral patients typically transferred to the referral facility? *Probe: In an ambulance, other vehicle, etc. How are they prepared?*
4. Approximately how much time does it take for the ambulance/other vehicle to arrive?
5. What information do you typically give to the ambulance or to the referral facility?
6. What coordination typically takes place with the receiving facility?
7. Can you describe your relationship with the nearest referral hospital?

**Work Environment/Motivation**

I would now like to ask you to answer several questions about your work environment. All answers are confidential. Please read the following statements about aspects of your work. For each of these aspects, please select the number that identifies whether you strongly agree, agree, are neutral, disagree or strongly disagree.

| **RESPONSE CODE** | |
| --- | --- |
| STRONGLY DISAGREE ……………………………… | 1 |
| DISAGREE………………….………………………….. | 2 |
| NEUTRAL (I.E. NEITHER AGREE NOR DISAGREE)… | 3 |
| AGREE …………………………….…………………... | 4 |
| STRONGLY AGREE ………………………………….. | 5 |
| NOT APPLICABLE …………………………………… | 96 |

|  | |  | |  |
| --- | --- | --- | --- | --- |
| **No.** | | **Item** | | **Response**  **(1 to 5)** |
|  | | In this facility, it is difficult to speak up if I perceive a problem with patient care. | |  |
|  | | I have the support I need from other personnel to care for patients. | |  |
|  | | The providers here work together as a well-coordinated team. | |  |
|  | | I feel comfortable asking for help from more experienced providers. | |  |
|  | | Our facility has a good relationship with the nearest referral facility. | |  |
|  | | For cases that require UBT, it is better to transfer them to a referral facility than to treat them here. | |  |

**Data Availability/Use**

1. What kinds of records does this facility keep for women delivering here?
2. What records do you keep about post-partum care?
3. How do you track information on PPH-related medication and supplies?
4. How does the facility use these records?

**Challenges**

1. What are the main challenges in the management of PPH in this facility?
2. How do you think PPH management in this facility could be improved?
3. In your opinion, what are some barriers to UBT use? ***SLOW DOWN***

*Alternative question*: What are some of the difficulties that you have experienced using UBT? What prevents providers from using UBT?

-*Probing Questions:*

1. Are there difficulties obtaining kits or supplies?
2. Is there a lack of training?
3. Are there religious, political, cultural, or institutional barriers?
4. Do you worry about other providers’/your patients’ perceptions of UBT?
5. Do you feel pressured to stick to the status quo?
6. Is there fear associated with using UBT?
7. Fear of what might happen if a woman dies and UBT was used?
8. Do you think that certain providers prefer other PPH treatments (e.g. hysterectomy)?
9. How do you think UBT use in this facility could be increased/improved? ***ASK WHY***

**Closing Remarks/Conclusion**

As we conclude our interview, we would like to ask you a few questions for closing remarks and comments.

1. Of all the things that we have discussed today, what is most important to you? What information would you like us to know?
2. Do you have any additional comments or questions?

Before we conclude the interview, is there anything you would like to add, or anything you would like to ask us? Thank you very much for taking time out of your day to speak with us!
