# Supplementary material for: “How I wish we could manage such things”: A qualitative assessment of barriers to postpartum hemorrhage management and referral in Kenya
Source: PLOS Glob Public Health. 2024 Nov 1;4(11):e0003842. doi: 10.1371/journal.pgph.0003842 (PMC11530065; doi:10.1371/journal.pgph.0003842)
Supplement: S2 Text — (PDF) [file pgph.0003842.s003.pdf]

Additional File S4 - Dictionary of codes

	Theme	Code	Subcode	Description
T1	Facility Infrastructure, Staffing, and Management	Facility staffing	-	Description of facility staffing systems, including potential shortage of staff
		Support from higher level providers	-	Description of availability or responsiveness from higher-level providers in response to emergencies
T2	PPH Management	Risk Factors for PPH	-	Description of how they typically screen for PPH / identify risk factors for PPH
		Monitoring for PPH	-	Description of monitoring actions (or lack thereof) for PPH, including typical procedures and specific PPH case
		Causes of PPH	-	Statement about the most common causes of PPH in their facility
		Identification of PPH	Methods for identifying PPH	Description of how PPH is typically identified in the facility
			Measuring blood loss	Description of how blood loss is measured (in general and for specific patient)
				Description of the process for estimating/Identifyng abnormal bleeding
		Medications and Supplies	Knowledge of medications and supplies for PPH	Description of the types of medictions and supplies that can be used for PPH
			Availability of medications and supplies for PPH	Description of availability of medicines and supplies for PPH Management
			Accessibility of medications and supplies for PPH	Description of accessibility of medicine and supplies for PPH management within the facility
T3	UBT Knowledge, Training, and Perceptions of UBT (device & procedure)	Knowledge of UBT	-	Participant statement on their own knowledge of UBT
		Perceptions of UBT	-	Participant's own perception of UBT
		Others' Perception of UBT	-	Participant statement on others' perception of UBT

T4	Referral Processes	Reasons for Referrals	-	Reason for referring the patient to a higher level facility
		Referral Processes & Decision-making	Referral decision-making	Description of facility-level roles and responsibiliries for making referral decisions
				Description of referral decision for specific PPH case
			Referral processes	Description of a typical referral process
		Referral Coordination	Exchanging Information	Description of information exchange between PHC and referral hospital
			Sub-county assistance with referral coordination	Descrtiptions of sub-county coordination or involvement with referral processes
		Relationships with referral facilities	Poor working relationships with referral hositals	Participant description of poor working relationship with referral facilities
			Good (or neutral) working relationships with referral hospitals	Description of good or neutral working relationship with referral facilities
		Delays in Referral Processes	Staffing delays	Description of delays in referrals due to staffing-related issues (e.g. provider decision-making, lack of staff, etc)
			Transportation delays	Description of delays in referrals due to transportation issues (e.g. no ambulance, transport is delayed, etc.)
			Faciliiy Identification Delays	Description of delays in referrals due to challenges in identifying a facility for referral
		Patient attitudes towards referral	-	Participant summary of patient attitudes towards referral
T5	Data Availability and Use	Reviewing PPH Case outcomes	Lack of formal review processes	Participant describes a lack of formal review processes for PPH cases they have managed
			Formal review processes	Participant describes facility-level efforts to review PPH managemnet
				Factors that participants note as challenges in managing postpartum hemorrhage
T6	Challenges to PPH management & Recommendations for	Challenges in PPH management	Knowledge & Training	Description of knowledge or training-related challenges to effective PPH management
			Supply	Description of supply-related challenges to effective PPH management
			Interpersonal/Staffing	Description of interpersonal or staffing-related challenges to effective PPH management

	improvements		Patient-related challenges	Description of patient-related challenges to effective PPH management
			Transportation to other facilities	Description of transprt-related challenges to effective PPH management
			No challenges in PPH management	Description of not perceiving any challenges to effective PPH management
		Improving PPH management	-	Recommendations for improving PPH management in their facility
		Barriers to UBT Use	Lack of experience or training	Description of lack of experience or training as a barrier to UBT use
			Supply	Description of Isupplies as a barrier to UBT use
			Acceptability of UBT	Description of the acceptability of UBT (device or procedure) as a barrier to UBT use
		Improving UBT use	-	Recommendations for improving UBT use in their facility
