# Supplementary material for: “How I wish we could manage such things”: A qualitative assessment of barriers to postpartum hemorrhage management and referral in Kenya
Source: PLOS Glob Public Health. 2024 Nov 1;4(11):e0003842. doi: 10.1371/journal.pgph.0003842 (PMC11530065; doi:10.1371/journal.pgph.0003842)
Supplement: S3 Text — (DOCX) [file pgph.0003842.s004.docx]

| **Cadre** | **N** | **Description of training required** |
| --- | --- | --- |
| Kenya Enrolled Community Health Nurse (KECHN) | *3* | These nurses hold a certificate (2 years of training). This is currently being phased out, and all are now required to be KRCHNs. Are most likely to be older and have more years of service. |
| Nurse-Midwife Kenya Registered Community Health Nurse (KRHN) | *8* | They have a diploma for their training (3 years of training) |
| Midwife (Senior enrolled nurse one)/ Senior Nursing officer | *3* | Have higher diploma in Midwifery. 2 years training post the diploma training. Have extensive professional experience. |
| ***Total*** | ***14*** |  |

*S3 – Breakdown of PHC Provider cadre*
