# Supplementary material for: “How I wish we could manage such things”: A qualitative assessment of barriers to postpartum hemorrhage management and referral in Kenya
Source: PLOS Glob Public Health. 2024 Nov 1;4(11):e0003842. doi: 10.1371/journal.pgph.0003842 (PMC11530065; doi:10.1371/journal.pgph.0003842)
Supplement: Attachment — Submitted filename: Miller_et_al_Response_to_reviewers2_15March24.pdf [file pgph.0003842.s005.pdf]

"How I wish we could manage such things": A qualitative assessment of barriers to postpartum hemorrhage management and referral in Kenya

Response to Editor (PGPH-D-23-02267)

15 March 2024

Dear Editor,

Thank you very much for your input on our manuscript. Please see below a response to your comments.

1. We note that you have indicated that data from this study are unavailable due to qualitative nature of data. We are grateful that you have provided us with the S1: PHC Provider Interview Guide. PLOS journals require authors to make all data necessary to replicate their study's findings publicly available without restriction at the time of publication. When specific legal or ethical restrictions prohibit public sharing of a data set, authors must indicate how others may obtain access to the data (https://journals.plos.org/globalpublichealth/s/data-availability#loc-acceptable-data-access-restrictions).

Before we proceed with your manuscript, please address the following prompts:
a) If there are ethical or legal restrictions on sharing a de-identified data set, please explain them in detail (e.g., data contain potentially identifying or sensitive patient information, data are owned by a third-party organization, etc.) and who has imposed them (e.g., a Research Ethics Committee or Institutional Review Board, etc.). Please also provide contact information for a data access committee, ethics committee, or other institutional body to which data requests may be sent.

We deeply value the principles of transparency and reproducibility upheld by PLOS. However, our study's unique context presents ethical challenges in sharing our qualitative data.

The intimate nature of our interviews with PHC providers, combined with the small sample size of 14 participants, heightens the risk of re-identification, despite de-identification efforts. This risk is compounded by the sensitive nature of the health information discussed, obligating us to prioritise participant confidentiality as per our IRB's guidelines, which states that : **"if data is shared outside of the certified research team, all personal identifying information will be removed, and responses will be anonymous and only aggregate information will be presented.".** *We only have permission to share aggregate responses.*

While we cannot make the full data set publicly available, we've included the S1: PHC Provider Interview Guide in our submission to facilitate an understanding of our research approach and the context of our findings. We are committed to

working within the ethical bounds to ensure our study's contributions are accessible and are open to discussing feasible alternatives for data sharing.

We appreciate your understanding of these constraints and look forward to finding a suitable solution that respects our participant privacy while contributing to the literature.

Contact:
Harvard T.H. Chan School of Public Health Office of Regulatory Affairs and Research Compliance 90 Smith Street, 3rd Floor
Boston, MA 02120
Protocol: IRB18-1342
Elizabeth Ehrlich: eehrlich@hsph.harvard.edu; cc: Margaret McConnell mmcconne@hsph.harvard.edu (PI)

Sincerely,
Junita Henry on behalf of the study authors
