# Supplementary material for: “How I wish we could manage such things”: A qualitative assessment of barriers to postpartum hemorrhage management and referral in Kenya
Source: PLOS Glob Public Health. 2024 Nov 1;4(11):e0003842. doi: 10.1371/journal.pgph.0003842 (PMC11530065; doi:10.1371/journal.pgph.0003842)
Supplement: Attachment — Submitted filename: Miller_et_al_Response_to_reviewers_7_data.pdf [file pgph.0003842.s006.pdf]

"How I wish we could manage such things": A qualitative assessment of barriers to postpartum hemorrhage management and referral in Kenya

Response to Editor (PGPH-D-23-02267)

16 September 2024

Dear Editor,

Thank you for your consideration of our manuscript. Please see below a response to your query.

a) Can you make a de-identified, anonymized, or aggregated data set publicly available in a repository or, if that is not possible, in your manuscript's Supporting Information files? For a list of recommended repositories, some of which are able to hold sensitive data, see here: https://journals.plos.org/plosone/s/recommended-repositories

b) Are there any institutional representatives who did not collaborate in the study and are not listed as authors on the manuscript who would be able to hold the data (the dictionary of codes) and respond to external requests for data access? If so, please provide their contact information (i.e., email address). Please also provide details on how you will ensure persistent or long-term data storage and availability. For instance, data might be stored in two independent locations.

We understand and support PLOS' commitment to data availability and transparency. However, as mentioned in previous correspondence, the nature of our study, involving interviews with healthcare providers regarding barriers to PPH management and referral in Kenya, raises challenges in ensuring both participant confidentiality and data security. Our interviews involved a small sample size (14 participants), discussing sensitive topics regarding the healthcare system in their region. The risk of re-identification remains a concern, even after anonymization or de-identification efforts. Additionally, following the confidentiality protocols required by the IRB, prevent the sharing of identifying or potentially identifying data outside of the research team. Therefore, we are unable to publicly share the full data set or provide a de-identified version that would meet both ethical obligations and the standards for protecting our participants' privacy. The context-specific nature of the interviews and small sample size, poses a risk of unintended identification of participants, even with anonymization.

We would like to propose that an author of the study be responsible for managing data access, due to their knowledge of the data, the ethical obligations to participants, and the already established IRB data protocols **(Junita Henry, junitahenry@g.harvard.edu).** We are also happy to include the **codebook dictionary** as Additional File S4: Dictionary of Codes.

Sincerely,
Junita Henry, on behalf of study authors.
